# mTOR inhibition triggers mitochondrial fragmentation in cardiomyocytes through proteosome-dependent prohibitin degradation and OPA-1 cleavage

**DOI:** 10.1186/s12964-025-02240-w

**Published:** 2025-05-31

**Authors:** Hugo E. Verdejo, Valentina Parra, Andrea del Campo, Cesar Vasquez-Trincado, Damian Gatica, Camila Lopez-Crisosto, Jovan Kuzmicic, Leslye Venegas-Zamora, Ursula Zuñiga-Cuevas, Mayarling F. Troncoso, Rodrigo Troncoso, Beverly A. Rothermel, Mario Chiong, E. Dale Abel, Sergio Lavandero

**Affiliations:** 1 Advanced Center for Chronic Disease (ACCDiS), Departamento de Bioquímica y Biología Molecular, Facultad de Ciencias Químicas y Farmacéuticas & Facultad de Medicina, Universidad de Chile, Santiago, Chile; 2 Departamento Enfermedades Cardiovasculares, Facultad Medicina, Pontificia Universidad Católica de Chile, Santiago, Chile; 3 SYSTEMIX Center for Systems Biology, O’Higgins University, Rancagua, Chile; 4 Facultad de Química y Farmacia, Pontificia Universidad Católica de Chile, Santiago, Chile; 5 Escuela de Química y Farmacia, Facultad de Medicina, Universidad Andres Bello, Santiago, Chile; 6 Departamento de Ciencias Biológicas y Químicas, Facultad de Ciencias, Universidad San Sebastián, Campus Los Leones, Lota 2465, Providencia, Santiago, Chile; 7 Instituto de Nutrición y Tecnología de los Alimentos (INTA), Universidad de Chile, Santiago, Chile; 8 Department of Internal Medicine, University of Texas Southwestern Medical Center, Dallas, TX USA; 9 Department of Medicine, David Geffen School of Medicine, University of California Los Angeles, Los Angeles, CA USA

**Keywords:** mTOR, Rapamycin, Mitochondrial fusion, OPA-1, OMA1, Prohibitin, AMPK

## Abstract

**Introduction:**

Cardiac mitochondrial function is intricately regulated by various processes, ultimately impacting metabolic performance. Additionally, protein turnover is crucial for sustained metabolic homeostasis in cardiomyocytes.

**Objective:**

Here, we studied the role of mTOR in OPA-1 cleavage and its consequent effects on mitochondrial dynamics and energetics in cardiomyocytes.

**Results:**

Cultured rat cardiomyocytes treated with rapamycin for 6–24 h showed a significant reduction in phosphorylation of p70S6K, indicative of sustained inhibition of mTOR. Structural and functional analysis revealed increased mitochondrial fragmentation and impaired bioenergetics characterized by decreases in ROS production, oxygen consumption, and cellular ATP. Depletion of either the mitochondrial protease OMA1 or the mTOR regulator TSC2 by siRNA, coupled with an inducible, cardiomyocyte-specific knockout of mTOR in vivo, suggested that inhibition of mTOR promotes mitochondrial fragmentation through a mechanism involving OMA1 processing of OPA-1. Under homeostatic conditions, OMA1 activity is kept under check through an interaction with microdomains in the inner mitochondrial membrane that requires prohibitin proteins (PHB). Loss of these microdomains releases OMA1 to cleave its substrates. We found that rapamycin both increased ubiquitination of PHB1 and decreased its abundance, suggesting proteasomal degradation. Consistent with this, the proteasome inhibitor MG-132 maintained OPA-1 content in rapamycin-treated cardiomyocytes. Using pharmacological activation and inhibition of AMPK our data supports the hypothesis that this mTOR-PHB1-OMA-OPA-1 pathway impacts mitochondrial morphology under stress conditions, where it mediates dynamic changes in metabolic status.

**Conclusions:**

These data suggest that mTOR inhibition disrupts mitochondrial integrity in cardiomyocytes by promoting the degradation of prohibitins and OPA-1, leading to mitochondrial fragmentation and metabolic dysfunction, particularly under conditions of metabolic stress.

**Supplementary Information:**

The online version contains supplementary material available at 10.1186/s12964-025-02240-w.

## Introduction

The average human heart beats approximately 100,000 times per day throughout our entire life. To fulfill this enormous task requires substantial energy generation, by mitochondria at a rate of nearly 6 kg of ATP daily. The mammalian target of rapamycin complex (mTOR), a signaling hub that senses nutrient abundance and regulates glucose and lipid metabolism, protein synthesis, and cell survival, plays a key role in regulating cardiac energetics. mTOR is inactivated under conditions of cellular stress or starvation as an adaptive response. Genetic deletion of mTOR in the heart causes dilated heart failure. Under sustained pressure overload, mTOR signaling is down-regulated, promoting mitochondrial autophagy [1, 2]. Despite the importance of both mTOR and mitochondria to cardiovascular health, the signaling pathways linking mTOR signaling and mitochondrial function in the heart are not fully understood.

Mitochondria form a functionally interconnected network, which is constantly remodeled through tightly regulated processes of fusion and fission that serve as a quality control mechanism, that restores respiration-impaired elements by excluding respiration-incompetent organelles for removal [1]. In the past, mitochondrial dynamics were thought to be limited in adult cardiomyocytes due to the ordered -- almost crystalline– disposition of mitochondria in close juxtaposition to sarcomeres. However, closer examination has shown that mitochondrial dynamics are critical in adult cardiomyocyte physiology, with cardiomyocyte mitochondria undergoing constant reorganization, particularly the cristae of the inner membrane [2]. In mammals, mitochondrial fission depends on the cytoplasmic GTPase dynamin-related protein 1 (DRP1), which can be recruited to mitochondria by several mitochondrial outer membrane proteins, namely fission protein 1 (FIS1), mitochondrial fission factor (MFF), mitochondrial dynamics proteins of 49 and 51 kDa (MiD49 and MiD51, respectively) [3]. Two separate types of protein machinery are involved in fusion; those responsible for the fusion of the outer membrane (MFN1 and 2) and the other for the fusion of the inner membrane (OPA-1). OPA-1 also plays a role in the regulation of cristae structure and the organization of respiratory complexes. OPA-1 levels are particularly high in the heart [4, 5]. The hearts of mice with heterozygous insufficiency (Opa-1^+/−^) contain abnormal mitochondria with reduced capacity for energy exchange with myofilaments and an increased hypertrophic response to pressure overload [6, 7]. Mutations in OPA-1 are associated with autosomal dominant optic atrophy in humans. Although no cardiac phenotype has been directly associated with OPA-1 mutations in humans, OPA-1 levels are reduced in heart failure, and electron micrographs of explanted hearts from transplant recipients reveal small and fragmented mitochondria, consistent with decreased fusion [8].

Despite the importance of OPA-1, many of its functions remain enigmatic. The protein has multiple isoforms, with both long and short forms being required for proper inner membrane fusion. Loss of electrochemical potential or low ATP levels in an individual mitochondrion leads to proteolytic processing of the long forms of OPA-1; if the accumulation of short isoforms goes unquenched, the affected mitochondrion is unable to fuse back to the network and is ultimately degraded through autophagy [9, 10]. Several proteases have been implicated in OPA-1 processing, including paraplegin, PARL, mAAA, and the zinc-dependent metalloprotease OMA1 [9–13]. Prohibitins (PHB) 1 and 2 are highly conserved proteins that perform distinct functions based on their intracellular localization. They are involved in stabilizing OPA-1 and cristae structure as well as in the maintenance of the mitochondrial genome [14] and regulation of fat metabolism [15, 16]. Recent studies demonstrate that inner mitochondrial membrane microdomains structured by PHB1/2 interact with OMA1, preventing its access to substrates, including OPA-1; thus, disruption of these microdomains can increase cleavage of OPA-1 with consequent morphological and functional changes in mitochondria [17, 18]. However, the upstream signals that control OMA1-mediated processing of OPA-1 are yet to be elucidated.

## Materials and methods

### Reagents

Antibodies against OPA-1 (Cat. ab42364), TOMM20 (Cat. ab186734), and PHB1 (Cat. ab1836) were purchased from Abcam (Waltham, MA, USA). Anti-mTOR (Cat. 2983), phospho-AMPKα (Cat. 2535), AMPKα (Cat. 2532), and K48-linkage specific polyubiquitin (Cat. 4289) antibodies were from Cell Signaling (Danvers, MA, USA). Mitotracker green FM (Cat. M-7514) and FBS were from Invitrogen (Waltham, MA, USA). Anti-DRP-1 antibody (Cat. 611738) was from Becton-Dickinson (Franklin Lakes, NJ, USA), whereas anti-FIS1 antibody (Cat. ALX-210-1037-0100) was from ENZO Life Sciences (Farmingdale, NY, USA). Anti-OMA1 antibody (Cat. 17116-1-AP) was purchased from Proteintech (Rosemont, IL, USA). AICAR (Cat. 2840), compound C (Dorsomorphin dihydrochloride, Cat. 3093) and Torin 1 (mTOR inhibitor, Cat. 4247) were purchased from Tocris Bioscience (Minneapolis, MN, USA). Anti-β-tubulin (Cat. T0198) and anti-GAPDH (Cat. G9545) antibodies, carbonyl cyanide m-chlorophenylhydrazone (CCCP, Cat. C2759), 2’,7’-dichlorofluorescein diacetate H2DCFDA (H2-DCF, DCF; Cat. D399), DMEM, M199 medium, rapamycin (Cat. R0395), o-phenanthroline (Cat. 107225), MG-132 (Cat. 474790) and other reagents were from Sigma-Aldrich Corp (Saint Louis, MO, USA). All the other inhibitors were from Calbiochem (San Diego, CA, USA). Protein assay reagents were from Bio-Rad (Hercules, CA; USA).

### Culture of cardiomyocytes

Cardiomyocytes were isolated from the hearts of neonatal Sprague–Dawley rats as previously described [19]. Rats were bred in the Animal Breeding Facility of the University of Chile. All studies conform to the Guide for the Care and Use of Laboratory Animals published by the US National Institutes of Health (NIH Publication, 8th Edition, 2011) and were approved by our Institutional Ethics Review Committee at the University of Chile (FONDAP 15130011). Primary cell cultures were incubated with or without rapamycin (100 nM) or AICAR (250 nM) for 0–24 h in DMEM/M199 (4:1) medium containing 10% FBS in the presence or absence of the different inhibitors and other genetic reagents. For the induction of starvation conditions, the DMEM/M199 medium supplemented with 10% FBS was replaced with RPMI which contains lower concentrations of nutrients and growth factors. RPMI provides essential vitamins, amino acids, and a moderate glucose concentration (2 g/L) but lacks the higher nutrient levels and metabolic substrates present in DMEM/M199. Additionally, DMEM/M199 formulations are optimized to support metabolically active and adherent cells by offering a more robust supplementation of amino acids, vitamins, and cofactors and the addition of 10% FBS provides growth factors, hormones, and proteins that enhance cell proliferation and survival. In contrast, RPMI offers more limited nutritional support, particularly for cells with high metabolic demands such as cardiomyocytes. RPMI contains a less supportive environment and under these conditions, AMPK activation was confirmed to validate the nutrient deprivation model, as we previously described [20–22].

### Cardiomyocyte transfection

Small interfering RNAs (siRNA) for Oma-1 and negative control (Mission, Sigma-Aldrich Corp.) and siRNA against Tsc2 (SignalSilence 6476, Cell Signaling) were used following the manufacturer’s instructions. The siRNAs used for knockdown experiments were as follows: a) Negative control, catalogue number SIC001 (Sigma-Aldrich Corp.); Oma-1, sense (5"GCCAUAAGAGAGGUCCGGA-3”) and antisense (5”-UCCGGACCUCUCUUAUGGC-3”). Briefly, the knockdown experiments were conducted using Opti-MEM supplemented with Oma-1 or Tsc2-specific siRNAs. RNAiMAX from Invitrogen Thermo Fisher (Cat. 13778150) was used as a transfection reagent. The knockdown of Oma-1 and Tsc2 were confirmed by Western blotting[23].

### Mitochondrial dynamics analysis

Cells were incubated for 30 min with Mitotracker green FM (400 nM) and maintained in Krebs solution. Confocal image stacks were captured with a Zeiss LSM-5, Pascal 5 Axiovert 200 microscope, using LSM 5 3.2 image capture and analysis software and a Plan-Apochromat 63x/1.4 Oil DIC objective, as previously described [ [5, 19, 24] Images were deconvolved with ImageJ, and then, Z-stacks of thresholded images were volume-reconstituted. The number and individual volume of each object (mitochondria) were quantified using the ImageJ-3D Object counter plug-in. Each experiment was done at least four times, and 16–25 cells were quantified per condition. An increase in mitochondrial volume and a decrease in the number of mitochondria were considered as fusion criteria [19, 24, 25].

### Immunofluorescence studies and colocalization analysis

Cells were fixed, permeabilized, blocked, and incubated with primary antibodies (anti-DRP-1 and FIS1 or TOMM20). Secondary antibodies were anti-mouse Alexa 456 for DRP-1 and anti-rabbit 488 for FIS1 or TOMM20. For the colocalization analysis, only one focal plane was analyzed. Images obtained were deconvolved, and the background was subtracted using the ImageJ software. Colocalization between proteins was quantified using Mander’s algorithm, as previously described [19, 26].

### ROS production

Once treatments were finished, cardiomyocytes were treated 10 min before cell lysis with 10 µM H2DCFDA in complete darkness. Cells were lysed with 100 mM NaOH and kept on ice, avoiding light exposure. Fluorescence was determined in cell extracts (excitation: 490 nm, emission: 525 nm). The lysates were always kept on ice until the assay was performed. Relative fluorescence was determined in all the experimental settings. Basal fluorescence was measured using cell lysates obtained from non-H2DCFDA preloaded cardiac myocytes (autofluorescence). The maximal fluorescence was determined using preloaded cell lysates obtained from H2DCFDA and hydrogen peroxide (1 mM) pretreated cardiac myocytes. As previously described, arbitrary fluorescence units were corrected for protein content determined by Bradford assay [27, 28].

### Oxygen consumption

Cells were plated on 60 mm Petri dishes and treated according to each experiment. Cells were then trypsinized, and the suspension (in PBS) was placed in a chamber at 25ºC, coupled to a Clark electrode 5331 (Yellow Springs Instruments) where the oxygen uptake was measured polarographically [5, 21, 29].

### ATP measurement

Intracellular ATP content was determined using a CellTiter-Glow ^®^ Luminescent Cell Viability Assay following the manufacturer’s instructions (Promega, Madison, WI, USA). Final luminescence was measured in a Top- Count NXT microplate luminescence counter (Perkin-Elmer, Waltham, MA, USA) as previously described [19, 29].

### Mitochondrial DNA quantification

As previously described, mitochondrial DNA (mtDNA) content was determined by quantitative real-time polymerase chain reaction (qPCR) [19, 30]. β-actin and mitochondrial cytochrome b were used as nuclear and mtDNA markers, respectively.

### Transmission electron microscopy

Briefly, cells were fixed in 2.5% glutaraldehyde in sodium cacodylate buffer, embedded in 2% agarose, post-fixed in buffered 1% osmium tetroxide, and stained in 2% uranyl acetate, dehydrated with an ethanol-graded series and embedded in EMbed-812 resin. Thin sections were cut on an ultramicrotome and stained with 2% uranyl acetate and lead citrate. Images were acquired on a FEI Tecnai G2 Spirit electron microscope equipped with a LaB6 source, operating at 120 kV as previously described [19, 24, 31]. Mitochondrial area and density, circularity, mitochondrial integrated density, and length were measured using the Multi-measure ROI tool of ImageJ (National Institutes of Health) software [19, 24, 31].

### Real time qPCR

The real-time PCR experiments were performed using SYBR green from Thermo Fisher Scientific (Waltham, MA). The data from each transcript was normalized to 18S using the 2ΔΔCt method [5, 23]. The rat primers used in this study were the following: mitochondrial SOD2 forward 5’CTGCTGGGGATTGATGTGTG-3’ and reverse 5’- CTACAAAACACCCACCACGG-3’; Catalase (CAT) forward 5’- ATAGCTGCCAAGGGAAAAGC-3’ and reverse 5’-ATTACTGGTGAGGCTTGTGC-3’; and 18 S forward 5’-CGGCTACCACATCCAAG-3’ and reverse 5’-CCAATGGATCCTCGTTA-3’.

### Western blot analysis

Once the designated treatment times were reached, the plates were washed three times with warm PBS. Cells were lysed with NP40 buffer supplemented with protease and phosphatase inhibitors. Homogenates were centrifuged at 21,600 x g for 3 min at 4 °C, and protein quantification was performed using the Bradford method (BioRad, Hercules, CA). The proteins were denatured in SDS buffer [19]. Equal amounts of protein were loaded and separated by molecular weight using SDS-PAGE electrophoresis. The polyacrylamide concentration was optimized based on the molecular weight of each target protein. The resolved protein samples were electrotransferred to PVDF membranes using a wet transfer system (400 mA for 90 min). The blocking agent was 5% non-fat milk dissolved in Tris-buffered saline containing 0.1% (v/v) Tween 20 (TBST). Membranes were incubated overnight with primary antibodies at 4 °C, followed by a 1 h incubation at room temperature with the respective peroxidase-linked secondary antibody. Chemiluminescence was detected with the Odyssey Fc Imaging System (LI-COR, Lincoln, NE) and densitometric analysis of the signals was carried out using the ImageJ software (NIH). Densitometric data were normalized to the values obtained for the loading control β-tubulin (β-TUB) or GAPDH.

### Inducible and cardiomyocyte-specific mTOR-deficient mice

Briefly, inducible and cardiomyocyte-specific mTOR-deficient [mTOR knockout (KO)] mice were generated by developing compound transgenic mice harboring a tetO-Cre construct (strain no. 6234, Jackson Laboratories), a reverse tetracycline transactivator under the control of the α-MHC promoter and floxed mTOR alleles (tetO-cretg/+/α-MHC-rtTAtg/+/mTORfl/fl). All mice were on a pure C57BL/6J background. To induce tetO-Cre expression, mTOR KO mice were administered doxycycline hyclate (DOX, Sigma, St. Louis, MO) at a 4 mg/kg body weight dose by intraperitoneal injection at 8 weeks of age. Then they were kept on doxycycline chow (1 g/kg) for 3 weeks, after which they were switched back to normal rodent chow for 1 more week to allow doxycycline washout before they were euthanized for experiments as has been previously described [32]. Expression of tetO-Cre results in recombination of the floxed mTOR alleles and deletion of the mTOR gene. mTORfl/fl mice were used as controls for mTOR KO mice. All experiments involving these animals were performed in accordance with protocols approved by the IACUC of the University of Utah and the Carver College of Medicine of the University of Iowa.

### PHB1 Immunoprecipitation

Immunoprecipitation of PHB1 was performed overnight using 2 µg of anti-PHB1 antibody (Abcam) on 400 µg of total protein. PHB1 was precipitated with Sepharose beads conjugated to protein G (A/G PLUS agarose, Cat. sc-2003, Santa Cruz), resolved by SDS-PAGE, and then polyubiquitination was assessed with anti-K48-linkage-specific polyubiquitin antibody.

### Statistical analysis

Statistical analyses were performed using GraphPad Prism version 6 software. Data are presented as the mean ± SEM. Unless otherwise specified, each experiment included at least three independent biological replicates (*n*). For statistical comparisons, a two-sided Student’s t-test or one- or two-way ANOVA was used, as appropriate, followed by Tukey’s post-test. A *p*-value < 0.05 was considered statistically significant. For in vivo animal experiments, a two-sided Mann-Whitney nonparametric test was used for data analysis. No inclusion/exclusion criteria were used. No animals were excluded from the analysis.

## Results

### mTOR inhibition promotes mitochondrial fragmentation and mitochondrial dysfunction in cultured cardiomyocytes

**Fig. 1:**
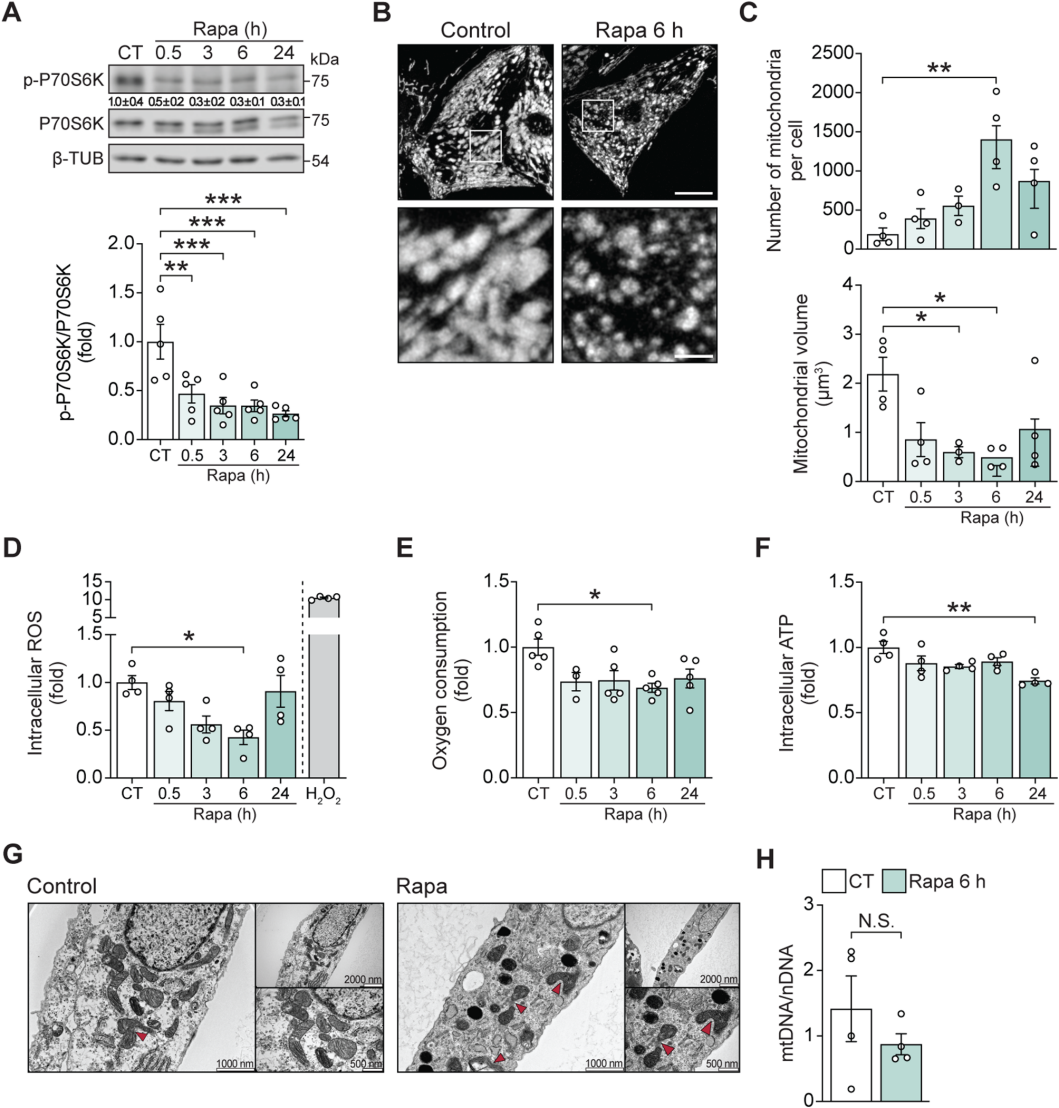
Rapamycin induces a mitochondrial fragmentation and dysfunction in cardiomyocytes. (**A**) Rapamycin reduces phosphorylation of p70S6 kinase. Total protein extracts were prepared from cardiomyocytes incubated with rapamycin (100 nM) for the indicated times. Phospho-p70 S6 kinaseThr389, p70 S6 kinase, and β-tubulin (TUB) levels were determined by Western blot. Representative Western blots are shown (*n* = 5). Relative quantification of phospho-p70 S6 kinaseThr389 bands (mean ± SD) is shown below the corresponding bands. (**B**) Primary cultured cardiomyocytes were treated with 100 nM of rapamycin for the indicated times and then loaded with mitotracker green (MTG, 400 nM). Images were obtained by confocal microscopy. Scale bar: 20 μm (*n* = 4); in the enlarged image, scale bar: 100 μm. (**C**) The number of mitochondria per cell and the relative mitochondrial volume were determined from cells in (B). (**D**) Mitochondria ROS production was determined in cardiomyocytes by H2DCFDA and flow cytometry after rapamycin (100 nM for 0–24 h) incubation. Hydrogen peroxide (H_2_O_2_, 1 mM) was used as a positive control (*n* = 4). (**E**) For oxygen consumption of cardiomyocytes treated with rapamycin, respiration was assayed by polarography using a Clark electrode under basal conditions (*n* = 5). (**F**) Intracellular ATP content in cardiomyocytes treated with rapamycin was determined using a luciferin-luciferase assay (*n* = 4). (**G**) Representative electron microscopy images of cardiomyocytes in control conditions and treated with rapamycin 100 nM for 6 h (*n* = 5). (**H**) Mitochondrial mass analysis using real-time PCR for mtDNA (*n* = 4). The data graphs correspond to the mean ± SEM. Each independent experiment is displayed as a dot in the graphs. Results were analyzed using one-way ANOVA followed by multiple Tukey’s comparisons. *P < 0.05 and ** *P* < 0.01

To study the regulation of mitochondrial dynamics and metabolism by mTOR signaling, cultured cardiomyocytes were treated with the mTORC1 inhibitor rapamycin, and changes in mitochondrial morphology and energetics were examined over time. Treatment with 100 nM rapamycin significantly reduced the phosphorylation of p70S6 kinase, a well-established downstream target of mTOR (Fig. 1A) [33, 34]. This was accompanied by a progressive increase in the number of mitochondria per cell, along with a reduction in the average volume of individual mitochondria (Fig. 1B–C). Mitochondrial energetics were also reduced in response to rapamycin, as evidenced by decreased oxygen consumption and reduced ROS production after 6 h of treatment (Fig. 1D–E). This coincided with increased mRNA expression of the antioxidant enzymes mitochondrial SOD2 and catalase (CAT), which likely contribute to the reduction in cellular ROS levels beyond the effect of decreased mitochondrial function alone (Suppl. Figure 1A–B). At later time points (24 h), a significant reduction in total cellular ATP was also observed (Fig. 1F). This last result is particularly interesting, as it contrasts with the earlier changes in mitochondrial morphology and the reduction in ROS levels. It likely reflects a rapamycin-induced shift in metabolic preference for energy production—from mitochondrial oxidative phosphorylation toward glycolytic pathways—triggered by mTOR inhibition [35, 36]. This shift may initially help compensate for impaired mitochondrial function but it appears insufficient to sustain the high energy demands of cardiomyocytes over prolonged periods, ultimately resulting in reduced cellular ATP levels.

Electron micrographs confirmed the presence of a fragmented mitochondrial phenotype 6 h after rapamycin treatment (Fig. 1G). However, total mitochondrial mass was apparently preserved as there was no change in mtDNA content (Fig. 1H). A different class of mTOR inhibitor, Torin 1, was used to confirm the rapamycin responses. Torin 1 competes with ATP for the active site of mTOR, acting as a potent suppressor of both mTORC1 and mTORC2 [37]. Treating cardiomyocytes with 100 nM Torin 1 (0–6 h) had the same effect on mitochondrial morphology and function as rapamycin (Suppl. Figure 2A-B), confirming mTOR inhibition as the mechanism of action through which rapamycin treatment triggers mitochondrial fragmentation in this model system.

### Rapamycin induces mitochondrial fragmentation through OPA-1 processing

**Fig. 2:**
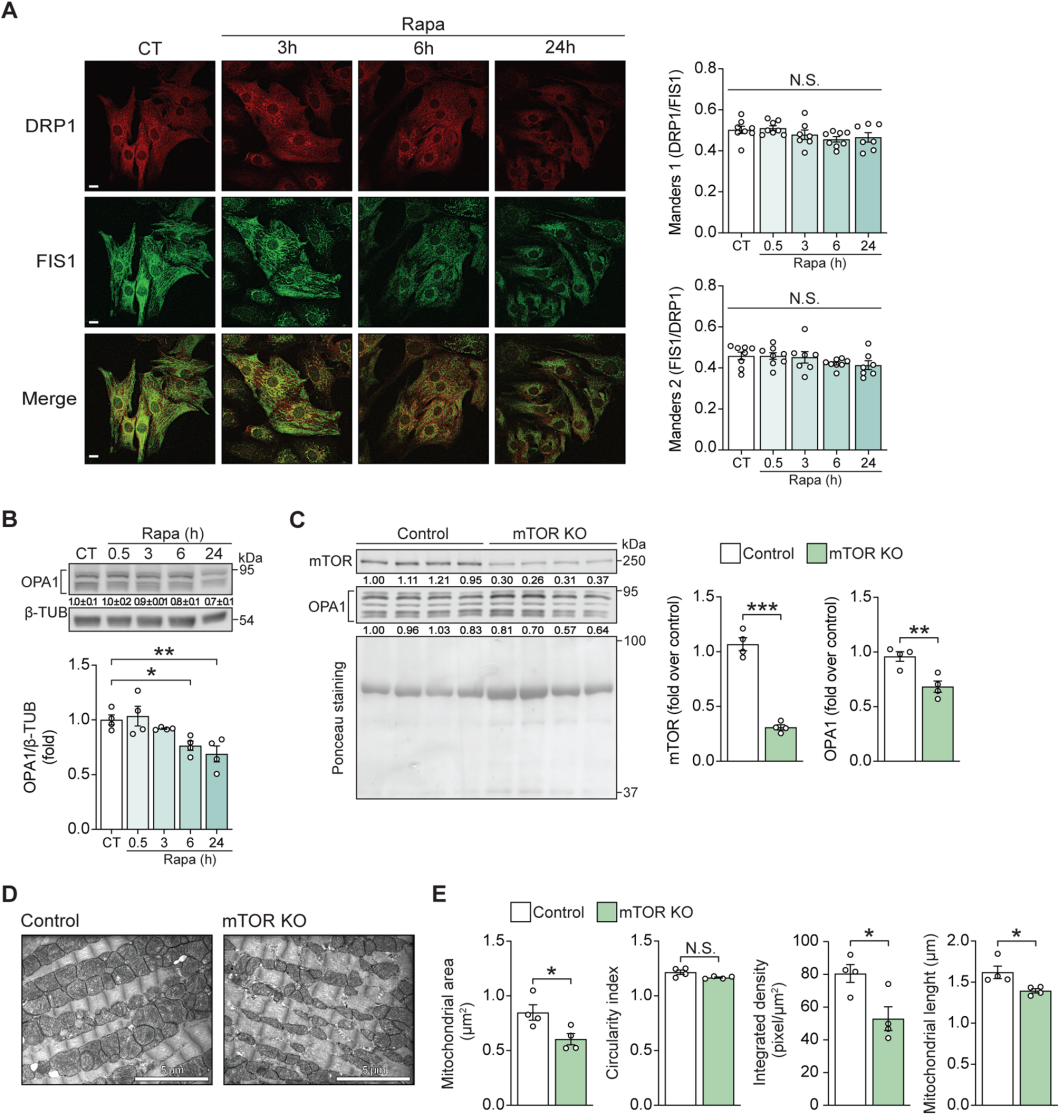
Rapamycin induces mitochondrial fragmentation through OPA-1 processing but not through DRP1 recruitment to the mitochondria. (**A**) Left: Control cardiomyocytes incubated with Rapamycin 100 nM for 0–24 h were immuno-stained for DRP-1 (red) or FIS1 (green) to determine colocalization (*n* = 8, 5–10 cells were evaluated in each time point per *n*). The scale bar is 10 μm. Right: Quantification of the effective colocalization of DRP-1 with FIS1. Rapamycin did not increase the effective colocalization of DRP-1 with FIS1, nor the effective colocalization of FIS1 with DRP-1. M1 and M2: Manders colocalization coefficients for DRP1 and FIS1, respectively. (**B**) Total protein extracts were prepared from cardiomyocytes incubated with Rapamycin (100 nM) for the indicated times. OPA-1 and β-TUB levels were determined by Western blot. Representative Western blots are shown (*n* = 4). Relative quantification of OPA1 (mean ± SD) is shown below the corresponding bands. (**C**) OPA-1 and mTOR protein levels in control and mTOR KO conditional mice treated with DOX. Hearts of control and DOX-treated animals were collected, and the proteins obtained were used for Western blot detection of OPA-1 (full protein, long and short isoforms) and mTOR. Ponceau-S was used as a loading control (*n* = 4). Relative quantification of mTOR and OPA1 (mean ± SD) is shown below the corresponding bands. (**D**) Electron micrographs of the left ventricular wall show disordered and fragmented mitochondria in the mTOR KO conditional mice treated with DOX compared to Control mice (scale bar: 5 μm). (**E**) The mitochondrial area, circularity index, cristae-integrated density, and mitochondrial length were quantified from the images in (D). Electron microscopy analysis data are from 100 mitochondria from each condition examined in four experiments. Scale bar: 5 μm (*n* = 4). The data graphs correspond to the mean ± SEM. Each independent experiment is displayed as a dot in the graphs. Results were analyzed using one-way ANOVA followed by multiple Tukey’s comparisons. * *P* < 0.05, ** *P* < 0.01, and *** *P* < 0.001

To define the molecular mechanisms downstream of mTOR inhibition that lead to mitochondrial fragmentation, we first examined the colocalization of the mitochondrial fission-related proteins FIS1 and DRP1. No increase in their colocalization was observed, suggesting that the fragmented phenotype was not due to enhanced DRP1-dependent fission (Fig. 2A). Moreover, colocalization of DRP1 with a general mitochondrial marker, TOMM20, was also assessed and yielded similar results (Suppl. Figure 3). We then looked for changes in the inner membrane fusion protein OPA-1 [1, 2], as we have previously reported an association between mTOR signaling and OPA-1[ [5, 38]. A significant decrease in the abundance of OPA-1 was evident 6 and 24 h after rapamycin treatment (Fig. 2B). OPA-1 is required for fusion of the inner mitochondrial membrane and the maintenance of mitochondrial cristae and the electron transport chain [39, 40]. Changes in OPA-1 levels and/or isoform distribution can lead to mitochondrial fragmentation and dysfunction. Using a cardiomyocyte-specific inducible mTOR KO mouse, we looked for in vivo evidence of mTOR control over mitochondrial dynamics [31]. Following doxycycline-induced KO of mTOR in the adult, there was a decrease in total OPA-1 protein in heart extracts (Fig. 2C), supporting a connection between loss of mTOR activity and decreases in OPA-1. Further, hearts from mTOR KO mice exhibited a reduced mitochondrial area, with no significant changes in the circularity index. A decreased integrated density was also observed, suggesting lower internal mitochondrial content, such as reduced cristae density. In addition, mitochondrial length was significantly decreased (Fig. 2D–E). Given that mTOR inactivation is a fundamental response to metabolic stress, these findings suggest that the mTOR/OPA1 pathway may contribute to the remodeling of mitochondrial structure and function under such conditions.

**Fig. 3:**
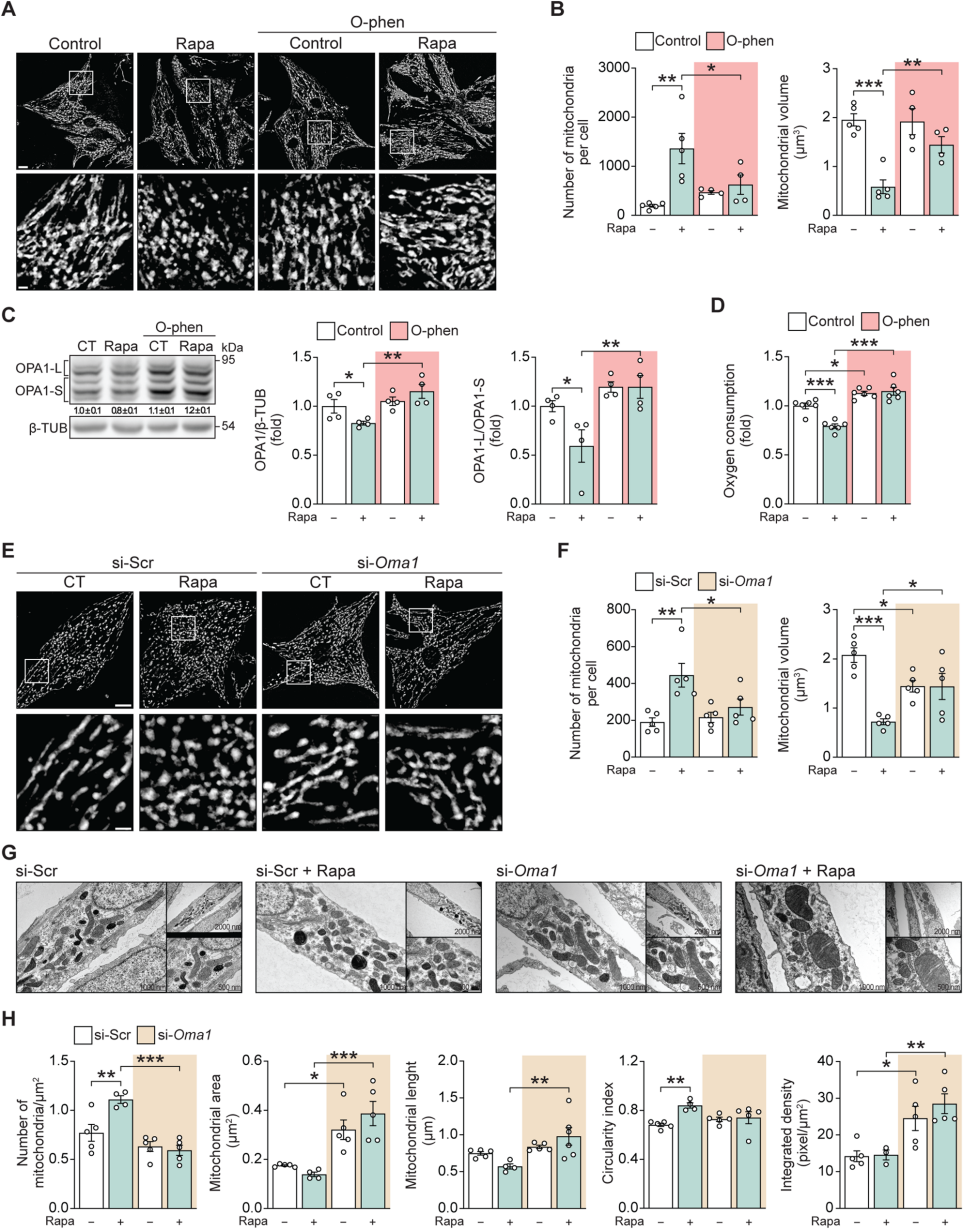
OPA-1 processing by OMA1 is responsible for the rapamycin-induced mitochondrial fragmented phenotype. (**A**) Primary cultured cardiomyocytes were treated with 100 nM rapamycin for 6 h and/or O-phenanthroline (O-phen, 10 nM) and then loaded with MTG (400 nM). Images were obtained by confocal microscopy. Scale bar: 10 μm (*n* = 5); in the enlarged image, scale bar: 50 μm. (**B**) The number of mitochondria per cell and the relative mitochondrial volume were determined from cells in (A). (**C**) Total protein extracts were prepared from cardiomyocytes incubated with rapamycin (100 nM, 6 h) and O-phen (10 nM). OPA-1 (total, large, and short isoforms) and β-TUB levels were determined by Western blot. Representative Western blots are shown (*n* = 4). Quantification of OPA1 (mean ± SD) is shown below the corresponding bands. (**D**) Oxygen consumption of cardiomyocytes treated with rapamycin (100 nM, 6 h) and O-phen (10 nM), was assayed polarographically using a Clark electrode under basal conditions (*n* = 6). (**E-F**) Mitochondrial morphology, the number of mitochondria per cell, and relative mitochondrial volume were obtained as described in (A-B) in cardiomyocytes stimulated with rapamycin (100 nM, 6 h) and pre-treated with a siRNA scrambled (si-Scr) or against Oma1 (si-Oma1). Scale bar: 20 μm (*n* = 5); in the enlarged image, scale bar: 100 μm. (**G**) Representative transmission electron microscopy images of si-Scr or si-Oma1 cardiomyocytes treated with rapamycin (100 nM) for 6 h. Three different magnifications of the same cell are shown. (**H**) The number of mitochondria per µm^2^, mitochondrial area, mitochondrial length, circularity index, and cristae-integrated density were quantified from the images in (G). Electron microscopy analysis data are from 100 mitochondria from each condition examined in four to five experiments (*n* = 4–5). The data graphs correspond to the mean ± SEM. Each independent experiment is displayed as a dot in the graphs. Results were analyzed using two-way ANOVA followed by multiple Sidak comparisons. *P < 0.05, **P < 0.01, and ***P < 0.001

Processing of OPA-1 is mediated by a variety of proteases, which can be inhibited pharmacologically by the metal-chelating agent O-phenanthroline (O-phen) [41, 42]. We tested whether a 30 min pretreatment with 10 nM Ophen altered the effect of rapamycin on OPA-1 processing and mitochondrial structure. Incubation with Ophen prevented rapamycin-induced mitochondrial fragmentation (Fig. 3A-B). It also prevented the decrease in total OPA-1 levels (Fig. 3C, left and middle panel) and the shift towards shorter OPA-1 isoforms (Fig. 3C, left and right panel), as well as the reduction in oxygen consumption (Fig. 3D). These data suggest that metalloproteases are involved in the OPA-1 and mitochondrial fragmentation response to rapamycin. In this regard, the increase in oxygen consumption observed in cells treated with Ophen is an intriguing finding. One possible explanation lies in the fact that neonatal cardiomyocytes exhibit a predominantly glycolytic metabolism. O-phen has been shown to enhance the activity of glyceraldehyde-3-phosphate dehydrogenase, thereby promoting aerobic glycolysis [43]. Additionally, inhibition of the metalloproteases responsible for OPA-1 processing results in increased availability of the long OPA-1 isoforms. This shift may improve mitochondrial cristae coupling, potentially leading to enhanced efficiency of oxygen consumption (Fig. 3D). Such an improvement could also correlate with a reduction in the generation of oxygen radicals, as previously reported for O-phen treatment [44]. These findings suggest a dual benefit of OPA-1 stabilization: improved mitochondrial function and reduced oxidative stress.

The mitochondrial metalloprotease OMA1, is integral to mitochondrial quality control and known for its role in modifying OPA-1 [41, 42]. To test for a dependence on OMA1, siRNA was used to deplete OMA1 from cardiomyocytes (Suppl. Figure 4A). Rapamycin increased mitochondrial fragmentation in cells treated with scrambled control siRNA (si-Scr), but not in Oma1-depleted cells (si-*Oma1*), as observed by confocal microscopy (Fig. 3E–F). However, when examined by electron microscopy, changes in the mitochondrial network were already evident in si-*Oma1* cells prior to rapamycin treatment, compared to si-Scr controls, likely reflecting the loss of basal OMA1 activity. This observation is consistent with our previous findings, where OMA1 knockdown alone was sufficient to stimulate mitochondrial fusion [5]. In this regard, mitochondrial area and integrated density were significantly higher in Oma1-depleted cells relative to controls (Fig. 3G–H). The rapamycin-induced increase in the number of mitochondria per area and in the circularity index observed in si-Scr cells (Fig. 3G–H) was suppressed in cells transfected with siRNA against Oma1. Additionally, mitochondrial length was significantly different between si-Scr and si-*Oma1* cells upon rapamycin treatment. These findings further support a model in which mTOR regulates mitochondrial morphology through a mechanism dependent on the mitochondrial protease OMA1.

### PHBs are essential for mitochondrial fragmentation under rapamycin treatment

**Fig. 4:**
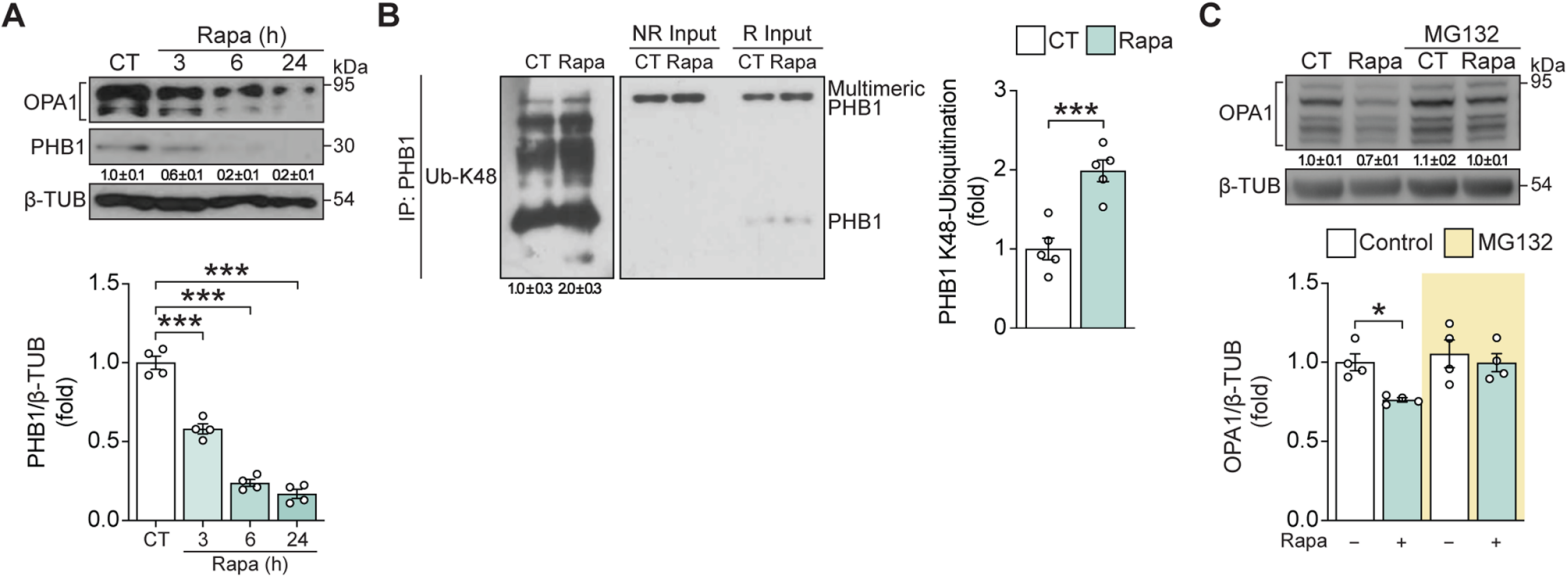
PHB1 ubiquitination contributes to OPA-1 processing mediated mitochondrial fragmentation by rapamycin. (**A**) Total protein extracts were prepared from cardiomyocytes incubated with rapamycin (100 nM) for the indicated times. OPA-1, PHB1, and β-TUB levels were determined by Western blot. Representative Western blots are shown (*n* = 4). Quantification of PHB1 (mean ± SD) is shown below the corresponding bands. (**B**) Total PHB1 protein was immunoprecipitated from total cell extracts of cardiomyocytes treated with rapamycin (100 nM) for 6 h, then probed with an antibody specific for anti-K48-linkage specific polyubiquitin antibody. Signal was normalized to total PHB1 (*n* = 5). Relative quantification of PHB1 Ub-K48 (mean ± SD) is shown below the corresponding bands. PHB1 in the total non-reduced (NR) and reduced (R) inputs is also shown. (**C**) Total protein extracts were prepared from cardiomyocytes incubated with rapamycin (100 nM, 6 h) and MG-132 (10 µM). OPA-1 and β-TUB levels were determined by Western blot. Representative Western blots are shown (*n* = 4). Relative quantification of OPA1 (mean ± SD) is shown below the corresponding bands. The data graphs are the mean ± SEM. Each independent experiment is displayed as a dot in the graphs. Results were analyzed using one or two-way ANOVA followed by multiple Tukey’s or Sidak comparisons, respectively. *P < 0.05 and ***P < 0.001

The PHB complex is an inner membrane, megadalton-sized scaffold composed of the highly conserved proteins PHB1 and PHB2 [45, 46]. Several functions have been identified for PHBs, including the stabilization of inner mitochondrial membrane microdomains that inhibit the interaction of OMA1 with its substrates, thereby stabilizing OPA-1 [18, 47]. OMA1 is known to cleave OPA-1 under conditions of stress [18, 48–51]. However, whether PHBs regulate OMA1 activity or OPA-1 stability in cardiomyocytes has not been studied. PHB1 levels were decreased in cardiomyocytes following rapamycin treatment (Fig. 4A). We postulate that the subsequent loss of PHB-associated microdomains and their repressive actions on OMA1 activity could underly the increased processing and degradation of OPA1 seen in cardiomyocytes following rapamycin treatment.

Ubiquitin-proteasome directed degradation of PHB mediates the removal of paternal mitochondria during fertilization [52]. In this regard, lysine 48-linked polyubiquitination of a protein functions as a degradation signal, targeting the modified protein for proteasomal degradation [53]. Co-immunoprecipitation analysis showed an increase in lysine 48-linked ubiquitination of PHB1 following rapamycin treatment (Fig. 4B), thereby implicating the proteasome in the loss of PHB1. To test whether a proteasomal-dependent step is required to increase the processing of OPA-1, cardiomyocytes were pretreated for 30 min with the proteasome inhibitor MG-132 (10 µM) prior to the addition of rapamycin. Figure 4C shows that MG-132 pretreatment prevented the rapamycin-induced decrease in total OPA-1 content. Furthermore, Suppl. Figure 5 shows that MG-132 also prevented the rapamycin-induced decrease in PHB1. These findings support a model where rapamycin increases mitochondrial fragmentation through an mTOR-PHB1-OMA1-OPA1 pathway under conditions of stress, involving the proteasomal degradation of PHB1, which releases OMA1 to act on OPA1, thereby altering mitochondrial structure. However, while our results suggest that the degradation of PHB1 and the release of OMA1 may at least indirectly contribute to this mechanism, we have only partially tested this and cannot exclude the possibility that other pathways may also play a role.

### Activation of AMPK by AICAR promotes mitochondrial fragmentation through the mTOR-PHB1-OMA1-OPA-1 signalling pathway

**Fig. 5:**
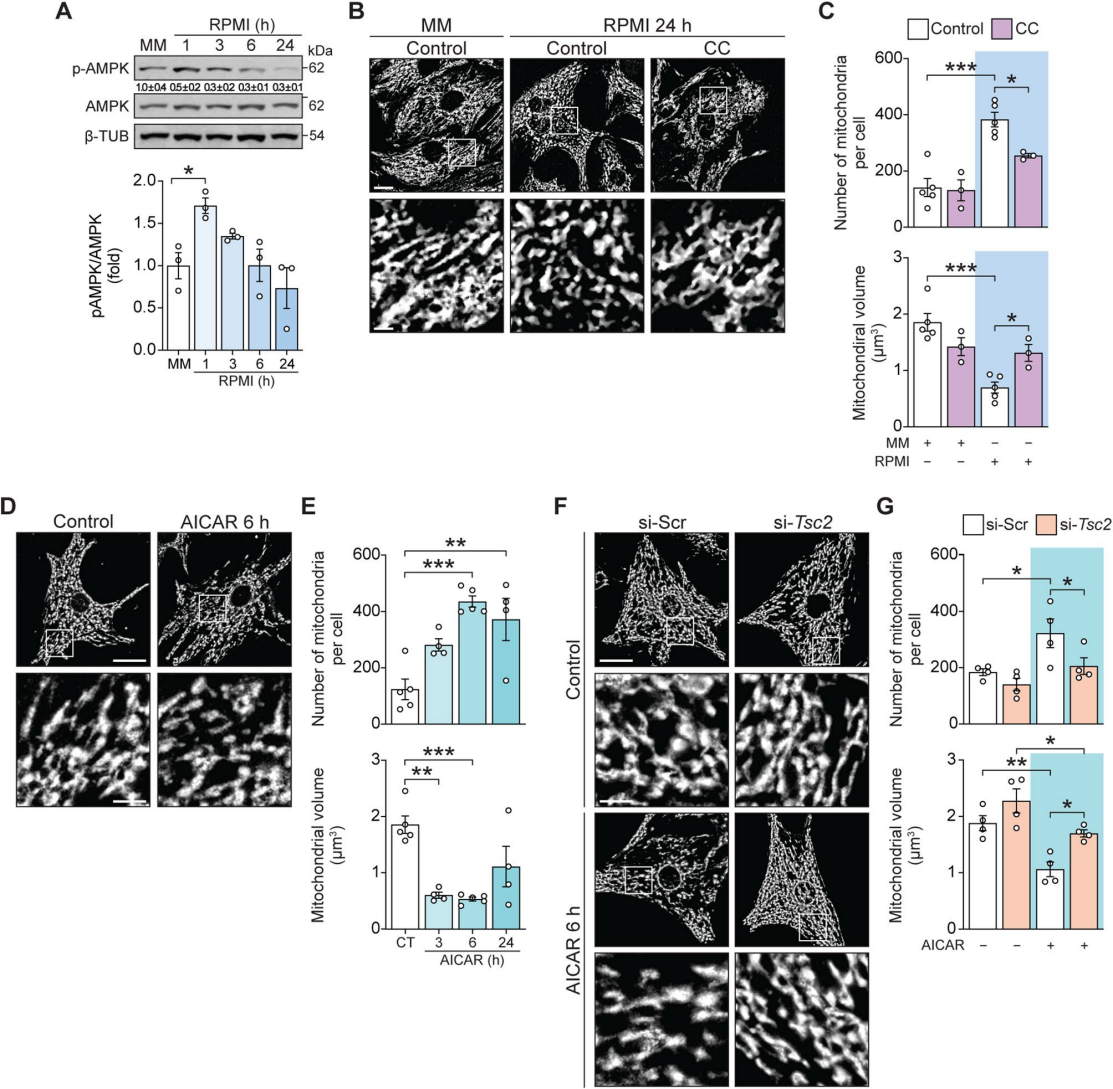
Nutrient deprivation with RPMI induces mitochondrial fragmentation through the AMPK-mTOR-TSC2 signaling pathway. (**A**) Total protein extracts were prepared from cells incubated with RPMI (0–24 h) for the indicated times. p-AMPK, AMPK, and β-TUB levels were determined by Western blot. Representative Western blots are shown (*n* = 3). Relative quantification of p-AMPK (mean ± SD) is shown below the corresponding bands. (**B**) Cardiomyocytes cultivated in RPMI for 24 h and/or pre-incubated with compound C (CC, 100 nM, 30 min prior RPMI) were loaded with MTG (400 nM). Multi-slice imaging reconstitution was obtained by confocal microscopy to show mitochondrial morphology. Scale bar: 10 μm (*n* = 5); in the enlarged image, scale bar: 50 μm. (**C**) Analysis of the number of mitochondria per cell and relative mitochondrial volume from the images described in (B). (**D-G**) Mitochondrial morphology, the number of mitochondria per cell, and relative mitochondrial volume were obtained as described in (B-C) in cardiomyocytes stimulated with AICAR (250 nM) for 0–24 h (*n* = 5) (D-E) and pre-treated with a siRNA scrambled (si-Scr) or against Tsc2 (si-*Tsc2*) (*n* = 4) (F-G). Scale bar: 20 μm; in the enlarged image, scale bar: 100 μm. The data graphs correspond to the mean ± SEM. Each independent experiment is displayed as a dot in the graphs. Results were analyzed using one or two-way ANOVA followed by multiple Tukey’s or Sidak comparisons, respectively. *P < 0.05, **P < 0.01, and ***P < 0.001

Mitochondrial dynamics and metabolism are inter-related [54]. Metabolic stress is frequently associated with changes in the structure of the mitochondrial network [54, 55]. Our results suggest that metabolic stress could impact mitochondrial dynamics through an mTOR-PHB1-OMA-OPA-1 signaling pathway. To investigate this further, we looked for activation of this pathway in response to nutrient deprivation, a more physiological stress condition that inactivates mTOR. Shifting cardiomyocytes to RPMI media leads to a rapid increase (1 h) in AMPK phosphorylation. Twenty-four hours later, mitochondria were smaller in size and more abundant than in control cells maintained in normal media, consistent with an increase in fission or decrease in fusion (Fig. 5A-B). Pharmacological inhibition of AMPK with compound C (CC, 100 nM, 30 min prior RPMI) suppressed fragmentation of the mitochondrial network in response to subsequent nutrient deprivation, as evidenced by no increase in the number of mitochondria per cell in the presence of CC and no decrease in the volume of individual mitochondria (Fig. 5C). Conversely, activation of AMPK, by treating with AICAR (250 nM) for 6 h was sufficient to cause a fragmented mitochondrion phenotype (Fig. 5D-E).

TSC2 is a GTPase activating protein (GAP) that inhibits mTORC1 by suppressing the activity of the small GTPase Rheb. Phosphorylation of TSC2 by AMPK increases its activity, thereby suppressing Rheb, and inhibiting mTORC1 [33, 34]. To determine whether AMPK promotes fragmentation of the mitochondrial network through the mTOR-PHB1-OMA1-OPA-1 signaling pathway, siRNA was used to deplete Tsc2 prior to treating with AICAR (Suppl. Figure 4B). siRNA depletion of Tsc2 from cardiomyocytes prevented mitochondrial fragmentation in response to AICAR (Fig. 5F-G), thereby supporting a role for mTOR inhibition and an mTOR-PHB1-OMA1-OPA-1 signaling cascade in the process of mitochondrial fragmentation induced by AMPK.

## Discussion

**Fig. 6:**
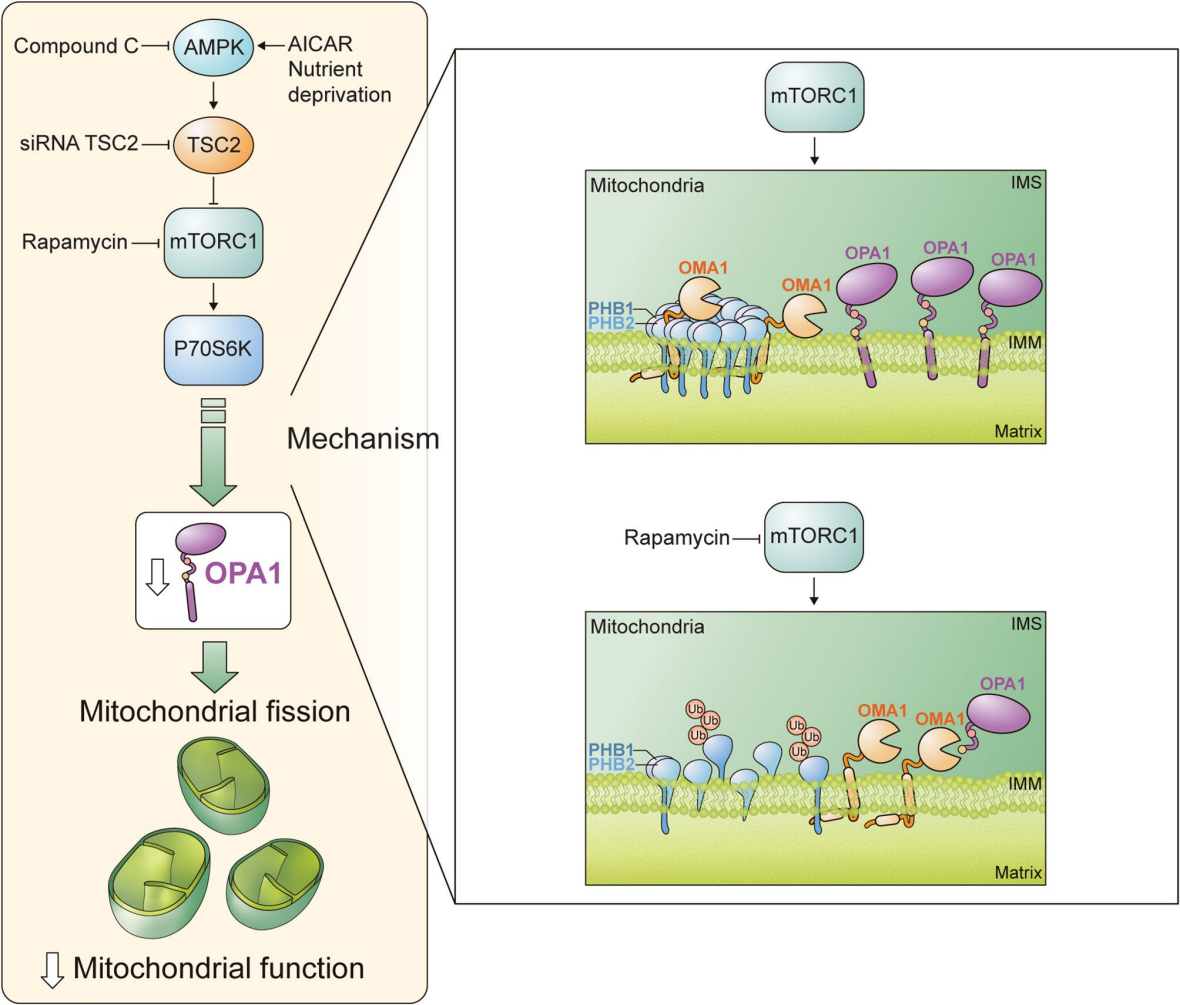
Working model. Pharmacological activation of AMPK promotes mitochondrial fission via the mTOR pathway, as siRNA targeting TSC2 reverses the mitochondrial changes induced by AICAR. AMPK activation through AICAR or nutrient deprivation impairs mitochondrial connectivity and function. Similarly, rapamycin-induced mTORC1 inhibition alters mitochondrial phenotype and reduces oxidative capabilities. Constitutively active mTORC1 (via TSC2 silencing) prevents AMPK-induced changes in mitochondrial morphology and function, highlighting mTOR’s key role in this pathway. Additionally, mTOR inhibition by rapamycin significantly reduces phosphorp70S6K levels and promotes mitochondrial fragmentation and dysfunction in cultured rat cardiomyocytes. Genetic interventions with siRNAs targeting Oma1 and TSC2 and mTOR knockout mice confirmed mTOR’s role in inducing mitochondrial fragmentation through OPA-1 processing. Rapamycin treatment also increased ubiquitinated PHB1 levels. Prohibitin proteins are crucial for mitochondrial fragmentation under rapamycin-induced stress, and this regulation of Prohibitins and mitochondrial morphology changes in cardiomyocytes is intricately linked to their metabolic status, driven by AMPK activation and mTOR signaling

Fully differentiated cardiomyocytes are highly metabolic muscle cells that need to respond and adapt to stressful conditions. Adult cardiomyocytes rely almost exclusively on mitochondrial ATP generation to meet their energetic needs [56, 57] Changes in mitochondrial morphology are intimately coupled to changes in cellular metabolism: mitochondrial fusion is usually associated with an increase in the oxidative capabilities of the organelle; while the converse is true for mitochondrial fission [2] Under conditions of energy depletion, as occurs in cardiac ischemia or heart failure, mitochondrial function is severely impaired [58, 59]. Although the mechanisms leading to mitochondrial failure are not fully understood, a common characteristic frequently noted is a decrease in the abundance of proteins involved in the control of mitochondria without significant changes in mRNA abundance, suggesting a non-transcriptional mechanism of regulation [60]. In contrast, much more is known about the molecular mechanisms involved in sensing and adapting to changes in metabolic demand; key among them is the AMPK- mTOR signaling pathway [61, 62]. Substantial experimental evidence suggests that activation of AMPK under stress conditions has a protective function in cardiovascular disease, regulating metabolism, autophagy, and endoplasmic reticulum stress responses [63]. AMPK activity has been shown to promote the fragmentation of the mitochondrial network through a variety of mechanisms. AMPK-mediated phosphorylation of MFF increases the recruitment of Drp1 to mitochondria [54, 64]. In C2C12 myoblasts, AMPK phosphorylation of MFF enhances mitochondrial fission and autophagosomal engulfment after TBK1 activation in a PINK1-Parkin-independent manner [65]. Here, our results provide evidence that AMPK activity promotes mitochondrial fission by inhibiting mTOR activity, as siRNA depletion of TSC2 blunts fragmentation of the mitochondrial network in response to the AMPK allosteric activator AICAR. Activation of AMPK, in response to AICAR or nutrient deprivation, reduced mitochondrial fusion and impaired mitochondrial energetics. Likewise, pharmacological inhibition of mTORC1, a signaling hub repressed by AMPK, caused a similar change in mitochondrial phenotype. TSC2 silencing abolished AMPK-dependent mTORC1 inhibition, thereby preventing the AMPK-driven alterations in mitochondrial morphology and function, and highlighting mTOR as the central regulator of this signaling axis (Fig. 6). These findings provide valuable complementary insights into the alterations observed in the cardiac-specific mTOR knockout model. In this model, mTOR deletion in the heart was achieved via doxycycline-induced overexpression of Cre recombinase, resulting in a significant reduction of mTOR protein (83%) and mRNA (91%) levels in cardiac tissue, as previously reported [32]. Although some residual mTOR activity remained, it was likely attributable to expression in non-cardiomyocyte cell types within the heart. Nevertheless, insulin-stimulated mTORC1 signaling was effectively impaired, as evidenced by decreased phosphorylation of S6 and 4E-BP1 [32]. Notably, mTOR expression in other tissues, such as liver and skeletal muscle, remained unaffected, underscoring the cardiac specificity of this model.

mTOR, a key metabolic regulator, has previously been shown to play a role in the control of mitochondrial dynamics, acting through a number of mechanisms, including modulation of mitochondrial fission and fusion events [66, 67]. mTOR activity promotes the transcription and translation of genes encoding mitochondrial proteins, thereby supporting mitochondrial biogenesis [68]. Suppression of mTOR activates autophagy, thereby facilitating the segregation of mitochondria and their degradation through mitophagy [69]. The relationship between mTOR and mitochondrial dynamics has been shown to be important in diverse cellular contexts, including neurodegenerative diseases, cancer, and metabolic disorders [70–72]. In neurodegenerative diseases, altered flux through mTOR signaling affects mitochondrial fission and fusion, contributing to disease pathogenesis [70]. Similarly, mTOR-dependent modulation of mitochondrial dynamics can influence cell transformation and metastasis in cancer [71]. In skeletal muscle, mTOR1 impacts mitochondrial dynamics during growth and hypertrophy [72]. In brown adipocytes mTOR regulates mitochondrial quality control to help maintain mitochondrial function and cellular phenotype [73]. Selective activation of mTOR in transgenic mouse models has been linked to improvements in cognitive deficits [66]. Our group previously demonstrated that treating cultured cardiomyocytes with insulin stimulates mitochondrial fusion and increases mitochondrial function by activating an Akt-mTOR-NFκB-Opa-1 signaling cascade [5]. Similarly, leucine supplementation has been shown to confer cardioprotection by enhancing mitochondrial function through a pathway dependent on mTOR and OPA-1 [74]. The present work advances our understanding of the mechanisms linking mTOR and OPA-1 in the regulation of cardiomyocyte mitochondrial dynamics and function.

Although mTOR inhibitors and AMPK activators are clinically used for different conditions, our findings suggest that both pathways converge on the regulation of mitochondrial dynamics through OPA-1. In this context, mTOR inhibition by rapamycin —approved by the FDA for the prophylaxis of organ transplant rejection, cancer, and certain autoimmune diseases [75]— has been primarily associated with its effects on autophagy. On the other hand, metformin, a first-line therapy for type 2 diabetes mellitus, activates AMPK indirectly by increasing the AMP/ATP ratio, thereby enhancing insulin sensitivity and reducing hepatic glucose production [76]. Beyond its metabolic effects, metformin also improves lipid metabolism, ultimately lowering cardiovascular risk in patients with diabetes [77, 78]. In cancer therapy, AMPK activation has been linked to the suppression of tumor growth. For example, AMPK activators such as metformin and AICAR have been shown to inhibit hepatocellular carcinoma proliferation via the LKB1–AMPK pathway [79].

AMPK also modulates key metabolic pathways in cancer cells, which often exhibit dysregulated energy metabolism [80]. Its activation has been shown to induce apoptosis in a variety of cancer cell lines, highlighting its potential as a therapeutic target for both cancer prevention and treatment [81]. Importantly, AMPK activators have also shown protective effects in cardiovascular disease. For instance, metformin confers cardioprotection against ischemia–reperfusion injury, likely through AMPK-mediated enhancement of autophagy and reduction of oxidative stress [82]. Furthermore, Zhang et al. [83], reported that a component of *Salvia divinorum*, a traditional Chinese medicine, protects against heart failure via AMPK/mTOR-mediated autophagy induction. This cardioprotective effect underscores the therapeutic potential of AMPK activators in heart disease, particularly in patients with diabetes who are at increased cardiovascular risk. Beyond metabolic and oncologic applications, AMPK activation also holds promise in renal disease. AMPK activators have been shown to improve outcomes in diabetic nephropathy and renal ischemia by reducing oxidative stress and inflammation, thus preserving kidney function [84, 85].

On the other hand, while PHB have diverse cellular functions, its primary role is thought to be mitochondrial [45]. PHB levels are high in tissues that rely heavily on mitochondrial energy metabolism. PHBs form alternating, heterodimeric ring-like complexes on the inner mitochondrial membrane that are required for mitochondrial stability [86]. The mitochondrial localized complexes are composed of both PHB1 and PHB2, and the loss of one protein leads to the loss of the other [45]. Recent studies by Coates et al. reported that in mammals, PHB levels decrease during cellular senescence [87]. Additional studies have shown that depleting either PHB1 or PHB2 results in disorganization of the mitochondrial PHB complex, mitochondrial dysfunction, and disruption of mitochondrial biogenesis [88, 89]. Zheng et al. demonstrated that the knockdown of PHB led to an increase in ROS production, reduced electron transport chain activity, and decreased ATP production. The resulting mitochondrial dysfunction contributed to the development of cardiac hypertrophy in cultured cardiac myocytes and in spontaneous hypertensive rats [90]. Conversely, overexpression of PHB has been shown to inhibit cell death under conditions of long-term exposure to hypoxia, suggesting that increased PHB function can be protective, improving cell survival [91]. Our current study provide evidence that rapamycin inhibition of mTORC1 causes fragmentation of mitochondria by promoting ubiquitination and degradation of PHB, leading to disorganization and loss of PHD-dependent membrane microdomains that prevent interaction of the zinc-dependent metalloprotease OMA1 with its substrates. This releases OMA1 for proteolytic processing of OPA-1, thereby reducing the capacity for mitochondrial fusion.

Also consistent with our findings, recent in vivo and in vitro studies have shown that PHB1 inhibits mitochondrial fragmentation and apoptosis in cardiomyocytes [92]. Furthermore, PHB1 has been implicated in other signaling pathways that promote mitochondrial fragmentation. Notably, PHB1 may interact with p53 and Bak, triggering mitochondrial fission in an OMA1-dependent manner [93]. Moreover, studies examining PHB-deficient mitochondria describe the presence of fragmented mitochondria with highly disorganized, swollen cristae and selective loss of the long isoforms of OPA1 (L-OPA1). These findings are consistent with L-OPA1 mediating mitochondrial fusion, potentially acting in concert with the shorter isoforms of OPA1 [45]. Similarly, PHB2 deficiency is reported to impair cardiac fatty acid oxidation and cause heart failure [94]. Taken together, our findings suggest a pivotal mechanism where the regulation of cardiomyocyte mitochondrial morphology is linked to their metabolic status through a dynamic interplay between mTOR and AMPK signaling, mediated through a mechanism involving PHB ubiquitination and activation of OMA-1-dependent OPA1 processing. Recognizing this critical crosstalk opens new avenues for therapeutic intervention, offering a promising direction for advancing our understanding of cardiac pathophysiology and developing new treatments.

## Electronic supplementary material

Below is the link to the electronic supplementary material.

Supplementary Figure 1: Effect of rapamycin on antioxidant enzymes mRNA expression. Primary cultured neonatal rat ventricular myocytes (NRVMs) were treated with 100 nM rapamycin for 0, 0.5, 3, 6, and 24h. mRNA levels of (**A**) SOD2 and (**B**) Catalase (CAT) were measured by RT-qPCR (*n* = 6). The data graphs correspond to the mean ± SEM. Each independent experiment is displayed as a dot in the graphs. Results were analyzed using one-way ANOVA followed by multiple Tukey’s comparisons. *P < 0.05 and **P < 0.01

Supplementary Figure 2: Torin induces mitochondrial fragmentation in cardiomyocytes. (**A**) Primary cultured cardiomyocytes were treated with 100 nM of Torin for the indicated times and then loaded with MTG (400 nM). Images were obtained by confocal microscopy. Scale bar: 20μm (*n* = 5); in the enlarged image, scale bar: 100μm. (**B**) The number of mitochondria per cell and the relative mitochondrial volume were determined from cells in (B). The data graphs correspond to the mean ± SEM. Each independent experiment is displayed as a dot in the graphs. Results were analyzed using one-way ANOVA followed by multiple Tukey’s comparisons. *P < 0.05 and **P < 0.01

Supplementary Figure 3: Rapamycin does not induce DRP1 recruitment to the mitochondria. (A) Left: Control cardiomyocytes incubated with Rapamycin 100 nM for 0–24h were immuno-stained for DRP-1 (red) or TOMM20 (green) to determine colocalization (*n* = 4, 12–20 cells were evaluated in each time point per *n*). The scale bar is 10μm. Right: Quantification of the effective colocalization of DRP-1 with TOMM20. Rapamycin did not increase the effective colocalization of DRP-1 with TOMM20, nor the effective colocalization of TOMM20 with DRP1. M1 and M2: Manders colocalization coefficients for DRP1 and TOMM20, respectively. The data graphs correspond to the mean ± SEM. Each independent experiment is displayed as a dot in the graphs. Results were analyzed using one-way ANOVA followed by multiple Tukey’s comparisons

Supplementary Figure 4: Effects of siRNA-mediated knockdown of Oma1 or Tsc2 in cardiomyocytes. Total protein extracts were obtained from cardiomyocytes treated with scrambled siRNA (si-Scr), siRNA targeting Oma1 (si-*Oma1*), or siRNA targeting Tsc2 (si-*Tsc2*) (*n* = 3). (**A**) OMA1 and GAPDH protein levels were analyzed by Western blot. Relative quantification of OMA1 (mean ± SD) is shown below the corresponding bands. (**B**) TSC2 and β-TUB levels were analyzed by Western blot. Relative quantification of TSC2 (mean ± SD) is shown below the corresponding bands. Data graphs are presented as mean ± SEM, with each dot representing an individual biological replicate. Statistical analysis was performed using one-way ANOVA followed by Tukey’s multiple comparisons test. *P < 0.05, **P < 0.01

Supplementary Figure 5: Proteosome inhibition prevents PHB1 degradation triggered by rapamycin. Total protein extracts were prepared from cardiomyocytes incubated with rapamycin (100 nM, 6h) and MG-132 (10 µM). PHB1 and β-TUB levels were determined by Western blot. Representative Western blots are shown (*n* = 6). Relative quantification of PHB1 (mean ± SD) is shown below the corresponding bands. The data graphs are the mean ± SEM. Each independent experiment is displayed as a dot in the graphs. Results were analyzed using a two-way ANOVA followed by multiple Tukey’s comparisons. *P < 0.05

Supplementary Figure 6: Uncropped Western blots. The uncropped Western blots for all the figures are presented here in the order of their corresponding figures and panels

## Data Availability

The main data supporting this study’s findings are available within the article or Supplementary Material; further inquiries can also be directed to the corresponding authors.
