# Supplementary figures and images for: mTOR inhibition triggers mitochondrial fragmentation in cardiomyocytes through proteosome-dependent prohibitin degradation and OPA-1 cleavage
Source: Cell Commun Signal. 2025 May 31;23:256. doi: 10.1186/s12964-025-02240-w (PMC12125784; doi:10.1186/s12964-025-02240-w)

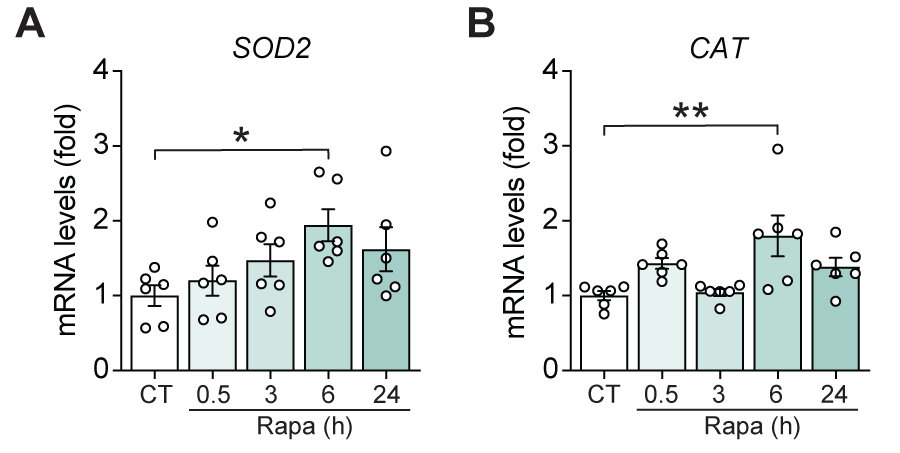

Supplement: Supplementary file 1 — Supplementary Figure 1: Effect of rapamycin on antioxidant enzymes mRNA expression. Primary cultured neonatal rat ventricular myocytes (NRVMs) were treated with 100 nM rapamycin for 0, 0.5, 3, 6, and 24h. mRNA levels of (A) SOD2 and (B) Catalase (CAT) were measured by RT-qPCR (n = 6). The data graphs correspond to the mean ± SEM. Each independent experiment is displayed as a dot in the graphs. Results were analyzed using one-way ANOVA followed by multiple Tukey’s comparisons. *P < 0.05 and **P < 0.01 [file 12964_2025_2240_MOESM1_ESM.png]

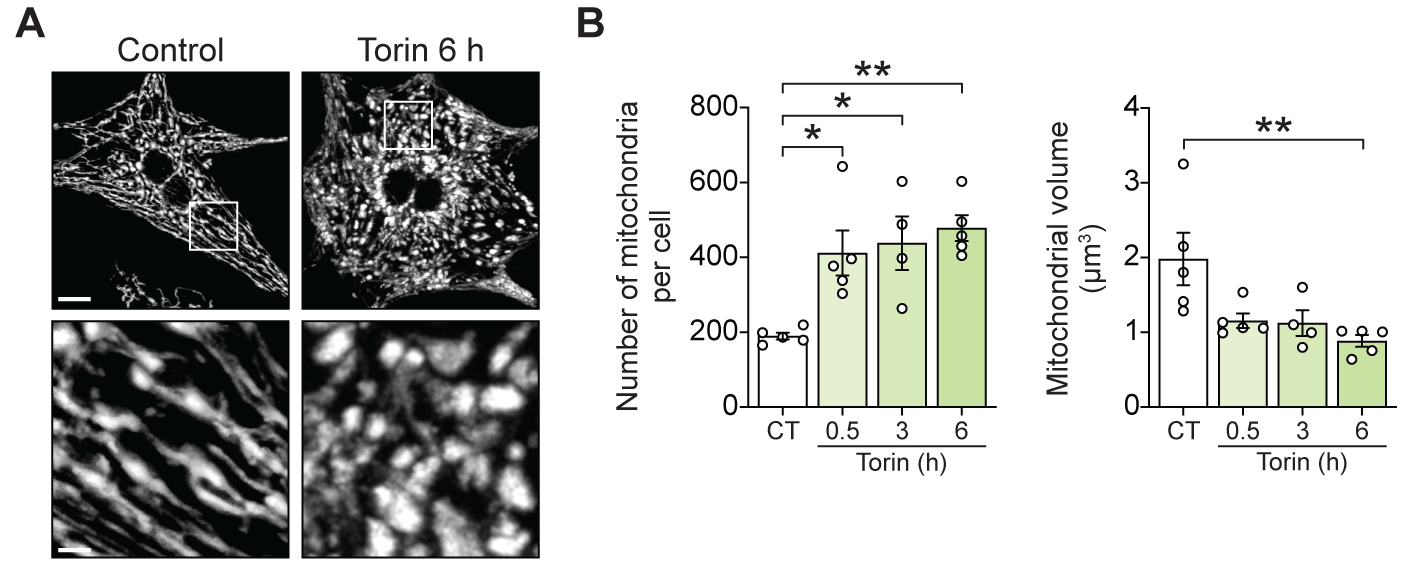

Supplement: Supplementary file 2 — Supplementary Figure 2: Torin induces mitochondrial fragmentation in cardiomyocytes. (A) Primary cultured cardiomyocytes were treated with 100 nM of Torin for the indicated times and then loaded with MTG (400 nM). Images were obtained by confocal microscopy. Scale bar: 20μm (n = 5); in the enlarged image, scale bar: 100μm. (B) The number of mitochondria per cell and the relative mitochondrial volume were determined from cells in (B). The data graphs correspond to the mean ± SEM. Each independent experiment is displayed as a dot in the graphs. Results were analyzed using one-way ANOVA followed by multiple Tukey’s comparisons. *P < 0.05 and **P < 0.01 [file 12964_2025_2240_MOESM2_ESM.png]

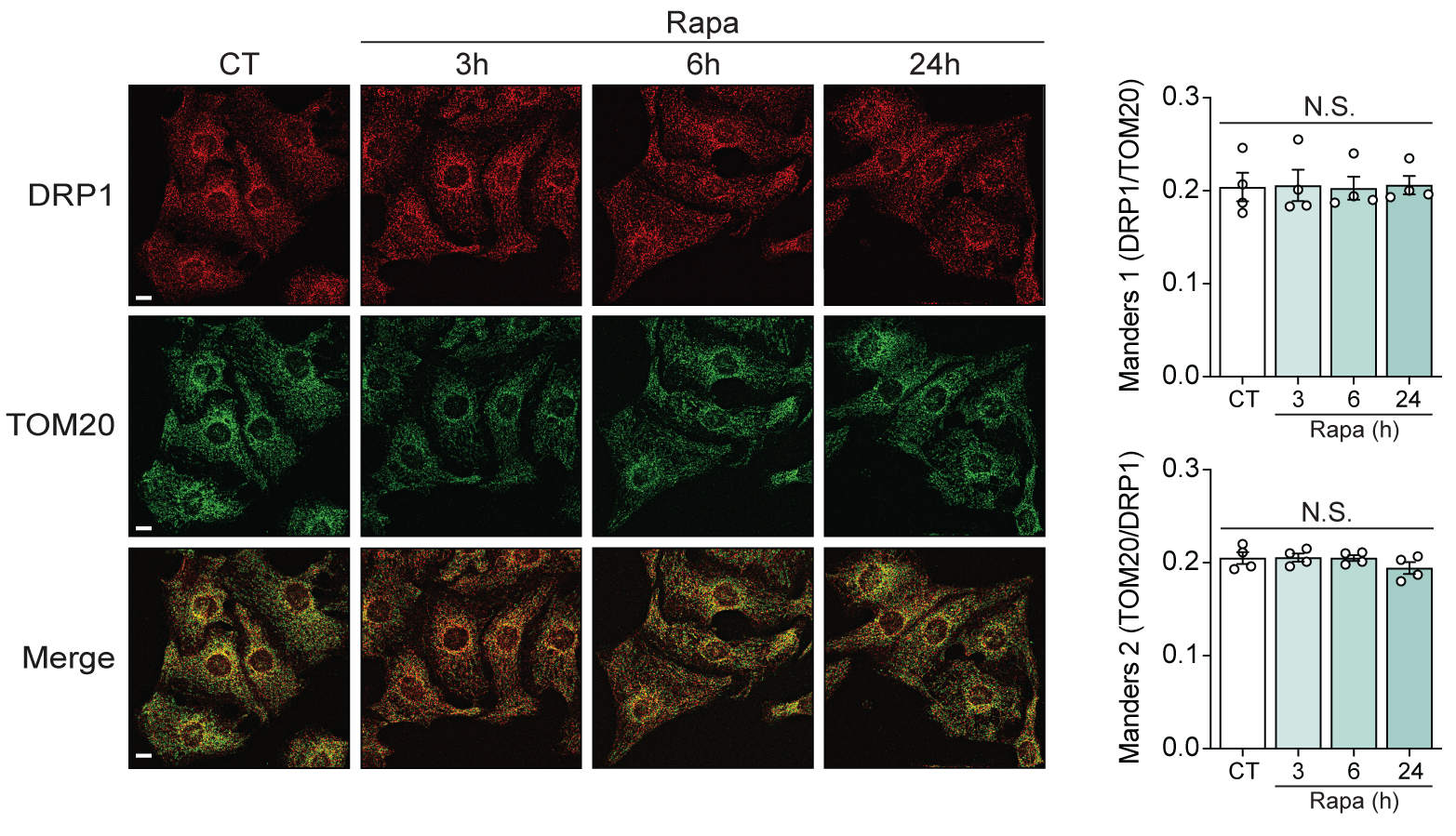

Supplement: Supplementary file 3 — Supplementary Figure 3: Rapamycin does not induce DRP1 recruitment to the mitochondria. (A) Left: Control cardiomyocytes incubated with Rapamycin 100 nM for 0–24h were immuno-stained for DRP-1 (red) or TOMM20 (green) to determine colocalization (n = 4, 12–20 cells were evaluated in each time point per n). The scale bar is 10μm. Right: Quantification of the effective colocalization of DRP-1 with TOMM20. Rapamycin did not increase the effective colocalization of DRP-1 with TOMM20, nor the effective colocalization of TOMM20 with DRP1. M1 and M2: Manders colocalization coefficients for DRP1 and TOMM20, respectively. The data graphs correspond to the mean ± SEM. Each independent experiment is displayed as a dot in the graphs. Results were analyzed using one-way ANOVA followed by multiple Tukey’s comparisons [file 12964_2025_2240_MOESM3_ESM.png]

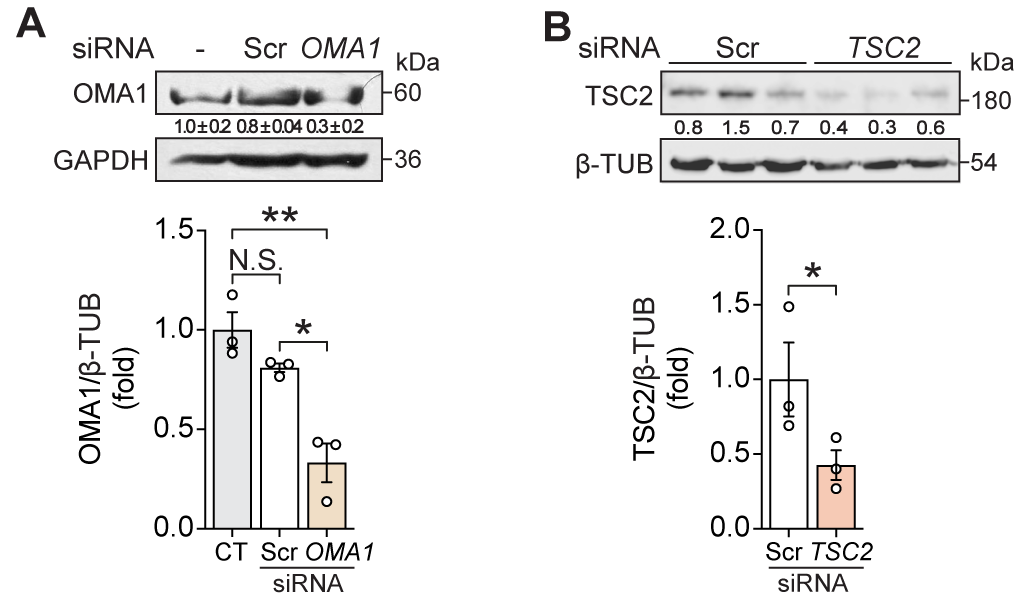

Supplement: Supplementary file 4 — Supplementary Figure 4: Effects of siRNA-mediated knockdown of Oma1 or Tsc2 in cardiomyocytes. Total protein extracts were obtained from cardiomyocytes treated with scrambled siRNA (si-Scr), siRNA targeting Oma1 (si-Oma1), or siRNA targeting Tsc2 (si-Tsc2) (n = 3). (A) OMA1 and GAPDH protein levels were analyzed by Western blot. Relative quantification of OMA1 (mean ± SD) is shown below the corresponding bands. (B) TSC2 and β-TUB levels were analyzed by Western blot. Relative quantification of TSC2 (mean ± SD) is shown below the corresponding bands. Data graphs are presented as mean ± SEM, with each dot representing an individual biological replicate. Statistical analysis was performed using one-way ANOVA followed by Tukey’s multiple comparisons test. *P < 0.05, **P < 0.01 [file 12964_2025_2240_MOESM4_ESM.png]

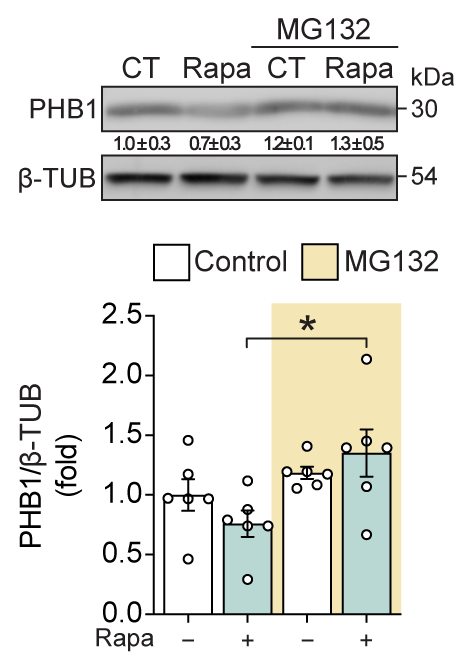

Supplement: Supplementary file 5 — Supplementary Figure 5: Proteosome inhibition prevents PHB1 degradation triggered by rapamycin. Total protein extracts were prepared from cardiomyocytes incubated with rapamycin (100 nM, 6h) and MG-132 (10 µM). PHB1 and β-TUB levels were determined by Western blot. Representative Western blots are shown (n = 6). Relative quantification of PHB1 (mean ± SD) is shown below the corresponding bands. The data graphs are the mean ± SEM. Each independent experiment is displayed as a dot in the graphs. Results were analyzed using a two-way ANOVA followed by multiple Tukey’s comparisons. *P < 0.05 [file 12964_2025_2240_MOESM5_ESM.png]

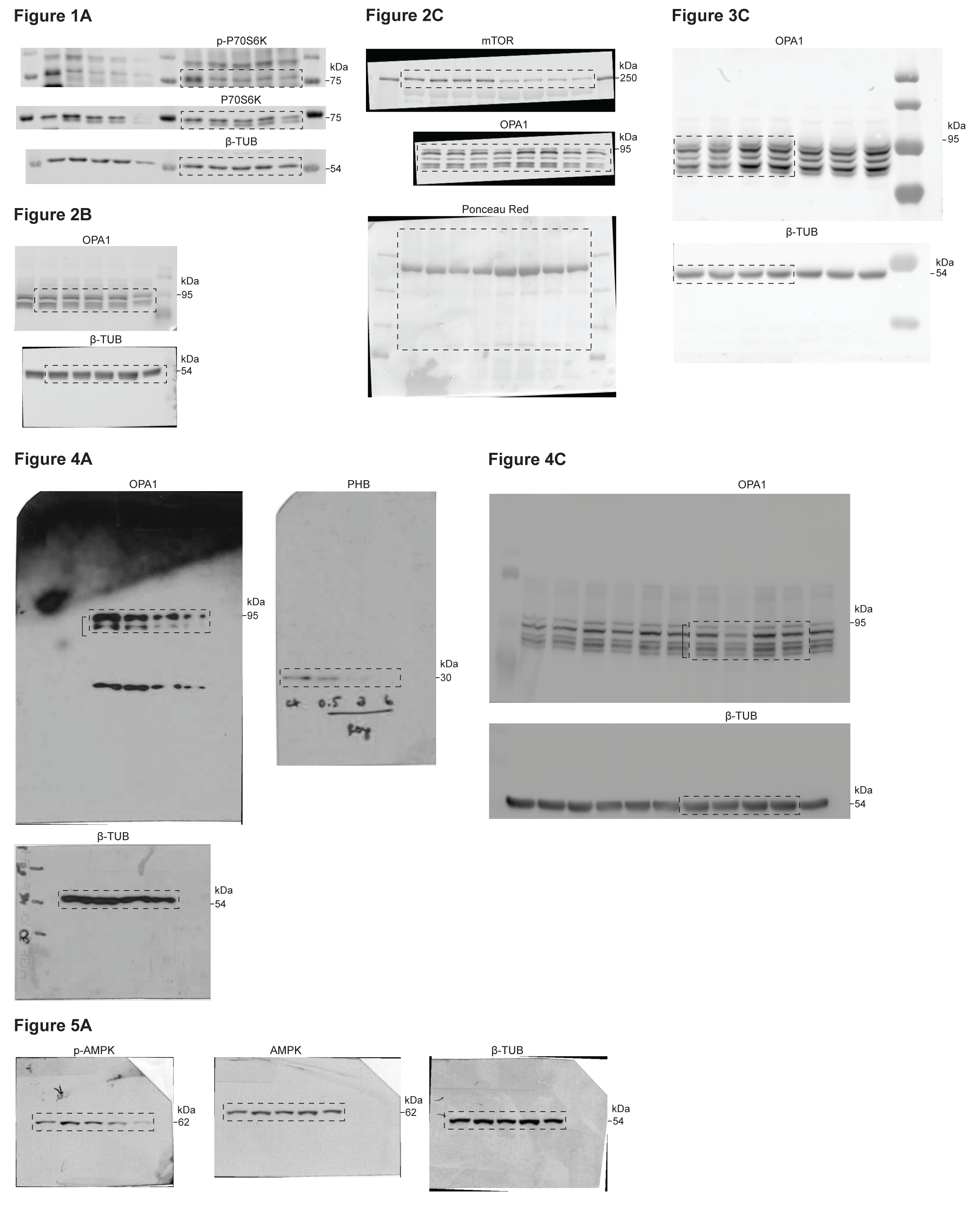

Supplement: Supplementary file 6 — Supplementary Figure 6: Uncropped Western blots. The uncropped Western blots for all the figures are presented here in the order of their corresponding figures and panels [file 12964_2025_2240_MOESM6_ESM.png]
